# Do corals dream of simulated seas?

**DOI:** 10.1007/s40656-025-00673-7

**Published:** 2025-05-27

**Authors:** Damien Bright

**Affiliations:** University of Chicago, 1155 E 60th Street, Chicago, IL 60637 USA

**Keywords:** Coral reefs, Evolution, Climate engineering, Climate critique, Time, Response-ability

## Abstract

What happens to a life science when its subject spans the globe yet appears fated to extinction? Such is the predicament that the field of international coral reef studies confronts under the strains of ocean stress. This article asks why this predicament becomes the basis for authorizing new powers of human intervention into the nature of biology. Through a genealogy and commentary of a theory and experiment known as “human-assisted evolution” and its quest for “super corals,” I examine the conceptual trouble that issues from calls to use corals to change global ocean change. I claim that the push to engineer marine life and worlds in response to ocean stress is as much an experiment in evaluating nature as it is in theorizing evolution. First, I offer the genre of “Big Coral” as a way of understanding a description of coral reefs as biological exemplars of global environmental change. Second, I offer a genealogical reading of human-assisted evolution as a whole Earth salvage operation grounded in a fantasy of geological time travel. Third, I locate the figure of the “super coral” and the trouble it raises not only in playing with the nature of corals but the nature of the human. I conclude with some reflections on ontological ambiguity that results from intervening in the nature of biology.

## Introduction

Why are the changing global oceans the kind of problem that calls for new powers of human intervention into the nature of nature? How are we to understand, for example, an asserted “need” to accelerate the evolution of marine life? Is such an acceleration tantamount to divining what particular life-forms want? This article engages these questions by investigating the shifting priorities and practices of a marine science field—international coral reef studies—as it seeks to reconstitute its naturally occurring research object—reef-building corals. Through a genealogical sketch of “assisted evolution,” a transnational research program to produce corals with the capacity to survive simulated future ocean conditions, I examine the conceptual trouble that arises from using corals to change ocean change.

My claim is that the push to engineer marine life and worlds in response to intensifying ocean stress is as much an experiment in evaluating nature as it is in theorizing evolution. Because, in order to conceptualize the whole Earth as something to salvage through technoscientific intervention, affective and practical reasoning get folded into biogeochemical descriptions. I support this claim in three parts, (1) showing how coral reefs come to embody global ocean change as a threat, (2) which motivates an experimental program to suspend coral reef collapse by embedding the human within their evolutionary future, (3) the examination of which puts a question mark over the nature of corals as well as the human. In contributing to burgeoning literature on the speculative bio- and geosciences, this article can, I hope, loosen a tendency within discussions of climate action that deem the engineering of life and worlds a special cause for deliberation, an abstraction of which to ask: “should we or shouldn’t we?” This very tendency, instead, follows from the way empirical descriptions of living nature are evaluative through and through yet massively strained under present conditions. Moral ambivalence about marine futures is not, then, something to argue away or domesticate with abstract rules but a crucial resource for grounding philosophical and political discussion, collaboration, and criticism.

Given that the present article considers an interventionist “turn” within coral science, it bears noting at the outset that history is replete with examples of people intervening in nature. Indeed, by some accounts, working up and working over the material world is definitional of the human form of life (Marx, 1981; Sennett, 2008). The mode of intervention I consider here is more specific. It is avowedly corrective and asserts that an advance in science and technology can treat a diagnosed malady in the metabolism of global nature by global society. It therefore resembles what scholars call the “technological fix,” wherein “technology is seen as the way out of a damaged environment and of a nature that shows its deficiency. The solution rests in the trust that humans can remake nature, in an ameliorated version” (Borghini et al., 2020, p. 122). There is, also, a history to the concept and critique of the technological fix. Often, such a fix is less a way of denying the sociopolitical complexity of a problem than subverting it by rationalizing questions of power, knowledge, and value by using the calculative languages of biochemistry, systems ecology, statistics and technics (e.g., hunger as a problem of calorie intake, carrying capacity, and crop yields). In response, scholarship on the technological fix emphasizes that how and why people solve problems—including with technoscience—can be just as philosophically puzzling and practically mired as what causes them (Scott, 2011). Hence, rather than an argument for the radical novelty of calls to intervene in nature, this article investigates the styles of reasoning at issue when such thinking is extended to the whole Earth. I do so through a close reading of one example of climate engineering for coral reef conservation.

Broadly speaking, the movement to intervene in the fundamental material processes of the biodynamic Earth is well underway and it is not obvious that anything will interrupt its trajectory (Kolbert, 2021; Masco, 2020b; Schäfer, 2023; Zee, 2020). Indeed, the frontier of coral reef intervention considered here is fast becoming a practical and regulatory heartland. Discussions of the “need” to redesign the biodynamic Earth are increasingly commonplace in mass media, international climate negotiations, and academic settings, stoked by capital injections from actors public, private, and somewhere in between. Such discursive and financial speculation tends to circulate a desire to sort the hypothetical from the happening and to separate “catastrophic” from merely “risky” efforts to intentionally alter the Earth’s fundamental biogeochemistry.^1^ The result is an atmosphere of anticipation that hovers over the global order of climate technoscience: When will human beings develop new powers to engineer life and worlds? When and where will they first get used? When will we learn of their side effects? Who will take responsibility for them? This atmosphere uncannily amplifies the existential anxiety thrown off by the very problem of climatic derangement that technoscience aims to address (Masco, 2017, 2020a). Thus, there may be something untimely, even indefensible, in treating the current conjuncture of climate crisis and responses to it as an occasion for more knowledge. To quote the name and rallying cry of one environmental organization founded in 1987: “Time’s Up!”.

Yet need all exercises in thinking environmental change be an attempt to get ahead of history’s curve? In different ways, conceptual history (Collingwood, 1960; Hadot, 2006), feminist technoscience studies (Colman, 2023; Haraway, 1989; Ruetsche, 2004), and psychoanalytic theory (Phillips, 2000) show that when we put questions and answers to the world we do not simply advance elective epistemological preferences. Rather, we experience and evaluate the world in one and the same move. Accordingly, we go on living by articulating intellect and sensibility in a way that draws in and draws out entrenched moral and political orientations. This dynamic is mysterious in its own right. Hence, and taking inspiration from scholars seeking ways “across” or “into” rather than “out” of a given problem (Berlant, 2007; Crary, 2016; Helmreich & Roosth, 2010; Hu, 2024), I ask: why have dying-but-not-yet-dead corals become ready fuel for a whole Earth salvage operation? How are we given to understand corals, oceans, and the human when technoscience troubles conceptions of species, habitat, and evolutionary time? I address these questions by examining a distinctive orientation within international coral reef studies, namely, an embrace of direct intervention into the lifeways of the organisms that build and give life to coral reefs. I do so via the example of “human-assisted evolution,” a research program to create predicted-but-as-yet-non-existent corals fit for predicted-but-as-yet-non-existent oceans. These entities are sometimes called “super corals.” First, I show how coral reefs have come to materialize global environmental change as a terminal condition through the genre I call “Big Coral” (Sect. 1.1). Then, I offer a reading of human-assisted evolution for corals as a mode of whole Earth salvage by examining its roots in terrestrial ecology (Sect. 1.2). Finally, I explain why an explanation of would-be super corals might be warranted. The entangled positing and proponing of “super corals” as vectors of whole Earth salvage, I submit, suspends a confrontation with human limitation that unassisted nature more readily invites (Sect. 1.3). I conclude by turning to the question of belated reasoning by way of a frequent touchstone in discussions of the limits of being, Philip K. Dick’s 1968 novel *Do Androids Dream of Electric Sheep?*

### Big coral, the genre and the generic

Within the broader domain of the contemporary marine sciences, international coral reef studies have an important yet somewhat ambiguous standing. Important because coral reefs are powerful proving grounds of bio-, geo-, and ecological inquiry that also pattern transnational encounters with and struggles over goods, ideas, and authority (Elias, 2019; Sponsel et al., 2015). Ambiguous because they are more territorially tethered than waves, tides, currents, marine food webs, fisheries, global weather circulation patterns, seabed structure and composition, or the marine carbon cycle—features that more readily index the oceans’ vast powers and availability as a Big Science concern (Adler, 2019; Helmreich, 2023; Rozwadowski, 2009). Yet in the late twentieth century, at the same time that genomic techniques recast the marine milieu as the ultimate laboratory for the study of life itself (Helmreich, 2009), tropical reefs and the corals that build them came to index nature’s limits. In what is frequently presented as a biological expression of an Earth climate increasingly given to extremes, the rapid degradation of coral reefs under pressure of ocean warming dramatizes the terrifying possibility of a coming mass extinction. I submit that this concerning development and its uptake by scientific and societal actors consolidates a genre of thought and action I will call Big Coral.

Big Coral is my way of referring to an orientation towards reef-building corals as vital ocean-going and thus Earth-defining entities, which points to a depth of significance beyond their involvement in the texture of people’s everyday lives. The scalar jump from tiny polyp to massive reef binds fragility to monumentality, making the reef-building coral a charismatic exemplar of life’s preciousness. I call Big Coral a “genre” in keeping with Timothy Morton’s (2018) use of the term to refer to how we bundle together ideas and attitudes when forming expectations of one another and the world. In this sense, we inhabit different genres when playing music, applying for work, seeking advice, or waking from a dream. The genre of Big Coral connects styles of biological reasoning with environmental appreciation in a double movement that, on the one hand, readily acknowledges the diversity of actually existing corals and reefs and the people they touch (including corals at lower latitudes or in the deep ocean that do not build reefs at all), while, on the other, leaning on a picture of tropical reef abundance to strategically emphasize coral’s singular status as foundational to the web of earthly life. When taken generically, corals do not just experience climatic derangement but portend its climactic denouement—mass extinction. Under such a description, they come to resemble sentinel devices, “living beings or technical devices that provide the first signs of an impending catastrophe” (Lakoff & Keck, 2013, p. 2). Yet, unlike the proverbial canary in the coalmine, the warning issued by particular corals builds in volume and reverb as it extends to reefs in general, the oceans, and the whole Earth. Big Coral therefore carries a moral charge, which lies not only in the sense of earthly oneness that results from using coral biology to transcend history and geography but also from the existential dread thus relayed.

To appreciate this demonstration, consider just the title of one widely circulated media report that is characteristic of the genre: “The Great Barrier Reef: a catastrophe laid bare” (Slezak, 2016). The colon in the title works like a hinge connecting a statement of fact with an evaluative invitation: reporting on the dramatic decline of one thoroughly regulated system of coral reefs is at the same time an existential confrontation with the Earth as endangered and therefore dangerous (Evans & Reid, 2015). Meanwhile, the wording reworks a standing trope within Occidental philosophies of nature, namely, that the physical universe is not merely puzzling but hides secrets for inquiring minds to reveal or “lay bare” (Hadot, 2006). Rather than an insight into the nature of nature that stokes a desire for further understanding, however, what lies exposed is existence as such—as in Agamben’s classic distinction between, on the one hand, bare life or “the simple fact of living” and, on the other, “the form or way of living proper to an individual or group.”^2^ And so, in a dramatic reversal, coral reefs no longer exemplify the metabolization of the Earth’s “potentialities” but their squandering. Put differently, Big Coral urges the troubling revelation that, soon enough, there may be nowhere for nature to hide.

The convergence of intellectual and affective judgment within the will to understand coral reefs is by no means a recent phenomenon. Indeed, tracking human-coral encounters can helpfully adduce internal relations between the science, history, and politics of nature across space and time (Bright, 2023; Helmreich, 2016; McCalman, 2014). Big Coral may be one such convergence, distinctive for elevating the prehuman history of corals, their whole Earth spread, and role in relaying climatic processes. The genre is identifiable across environmental media coverage (Jacobsen, 2016), scientific surveys (Hughes et al., 2017), global environmental organizing (McCalman, 2017), and environmental humanities scholarship (Braverman, 2018; Schuster, 2020). Thus, as a natural historical shorthand, Big Coral leans on a generic understanding of corals as lifegiving and worldmaking in a way that can minimize and even indict human exceptionalism. Resonant with the “declensionist” Occidental tradition of end-of-nature discourse (Merchant, 2003), Big Coral is nevertheless scaffolded upon the secularizing geological concept of “deep time” and its ceaseless measurement (Hu, 2025; Rudwick, 2014). For this reason, the genre invites a degree of ambivalence towards present conditions: coral reefs index a present-day reality of ocean change, whereby heat waves and thermal shock are at once generalized and uneven, yet at the same time they suggest that far graver changes are just over the horizon.

Though the genre waxes tragic, Big Coral is not necessarily defeatist. Indeed, this way of inhabiting global environmental change resembles what humanities scholars have lately referred to as the need to adopt a “geohistorical” or a “planetary” perspective (Chakrabarty, 2009, 2019). In this sense, Big Coral can be a way of imagining extinction that raises “questions of what we value and what stories we tell” (Heise, 2016, p. 5). Yet who—or perhaps when and where—is this “we”? As I understand it, the critical force of Big Coral lies in refracting a whole Earth gaze, which suggests a corresponding generalized humanity, united in existential danger (however prospectively) and by a lack of common leadership (however contingently). Indeed, the plight of corals is often taken as a proverbial sign of the times, a symptom of a current planetary epoch in which humankind has wrecked the planet and must, whether under the description of the Anthropocene or the Capitalocene or an equivalent world-historical category, remake itself (J. Moore, 2016). Thinking with Big Coral can therefore overlook or downplay ways of living with corals that do not point to the same universal horizon or overcoming—for example, everyday lives, negotiations, and ambivalences of coral reef use, care, and dependency (Klein, 2017; Rivera-Sotelo, 2024).^3^

We can acknowledge that using the generic lifeways of reef-building corals to convey the urgency and complexity of ocean stress to so many (including millions who may never so much as see a coral reef with their own eyes) is a remarkable achievement of “science communication” while appreciating, at the same time, the possible confusion it creates: that the condition of reef-building corals will be mistaken for the cause of ocean stress. Big Coral can focus a desire for new powers of conservation that would amount to a whole Earth salvage operation: a heroic intercession in the unraveling of a presumed chain reaction connecting the fate of corals, the oceans, and life itself (Moore, 2021). Put directly, Big Coral can motivate intervention into the generic lifeways of reef-building corals as a mode of climate crisis resolution. Shortly, I will discuss how this arises in terms of the theory and experiment of human-assisted evolution. But first, let me draw you into the genre more fully to appreciate the way reef-building corals participate in and relay climatic derangement as a matter of marine biogeochemistry.

Coral reefs are frequently likened to rainforests or cities because they support complex lifeways by way of dynamic relations with their surrounds. Wave friction, bathymetric pressure, storm impacts, the feeding and nesting habits of myriad creatures—all this and more condition patterns of reef growth and decay. This arrangement has local dimensions, for single corals, and global dimensions, for entire reefs. Moreover, it enfolds human and more-than human communities in recursive coexistence, which means that reefscapes sustain food ways, orient migration flows and provide breakwaters for coastal protection such that corals, in more humanistic terms, are the stuff of home, hearth, and heritage. The growth forms of coral reefs provided crucial inspiration for Charles Darwin’s theory of evolution and, a century later, provided the material evidence to support its account of so-called deep time (Bredekamp, 2008; Sponsel, 2018). Paleoecologists have used fossils to reconstruct a geological record of coral evolution as far back as the Cambrian period. Corals are constantly growing skeletons of calcium carbonate, at a rate that varies according to surrounding conditions, which can then be read much like tree rings. Hence, as with other “Earth indices” like pollen and ice, coral cores archive biogeochemical variations over many hundreds of years that bespeak climate trends (Lough & Cooper, 2011; McKean, 2024; Rosol et al., 2023).

Earlier, I noted that not all corals are reef-builders. It goes the other way too: not all reefs are coral-built. For many millions of years, sponges were the dominant reef-builders until a change in ocean conditions drastically reduced their number. Other marine organisms did so before them. In fact, it turns out that major extinction events are accompanied by “reef gaps” that can stretch from one to ten million years. During this time, reef-building organisms and their works effectively vanish from the geological record of Earth’s deep history only to reemerge in new combinations (Veron, 2008). From one mass extinction to the next, it takes time for an organism functionally capable of building reefs to evolve and set about its labors. There are rival theories of explanation for why this is. But what “reef gaps” show is that the internal relation between today’s reef-building corals, oceans, and Earth holds since the Cretaceous-Tertiary mass extinction (66 million years ago) but there is a different order of internal relation between the reef form and the reef-building biological entity across mass extinctions.

To some extent, coral reefs can weather disruption. They can recover from seasonal variability, storm impacts, fishing, agricultural runoff, coastal development, etc. Indeed, the history of international coral reef studies is, in part, the history of making such “encroachments” available to “management” and thereby stabilizing an ecological abstraction of coral reefs as locally variable yet globally distributed indicator of human industrialization (Stoddart, 2001). Toxicity and eutrophication continue to harm reefs, yet compounding these more-or-less localized threats are the scale and pace of ocean change, which puts a global question mark over the future of reef-building corals. Current coral science gives three principal reasons: (1) corals cannot regulate their body temperature, so fluctuating and gradually increasing water temperatures compromise their basic metabolic processes; (2) as carbon dioxide concentrations increase in the ocean so does acidity, which reduces the amount of carbonate ions available for corals to build their skeletons and so compromises reef integrity; (3) higher temperatures and light levels strain the crucial symbiosis between coral polyp hosts and the endosymbiotic algae who sustain them through photosynthesis (Gates et al., 1992; Hoegh-Guldberg, 1999). This relationship goes from sympatric to toxic when symbionts produce more oxygen than their hosts can consume. A breakdown results; host and symbiont part ways. Without onboard algae, corals become translucent and expose their underlying calcium carbonate skeleton. This microphenomenon is visible at the macroscale because, like other critical biological processes such as annual reproduction, it is one that corals across species perform in synchrony. The expression “mass coral bleaching” names this population-scale loss of color and vitality.

As a mass phenomenon, bleaching puts coral reefs under considerable physiological and reproductive strain. Reefs can recover from localized and occasional bleaching thanks to species diversity, larval resettlement, and biological adaptation. Yet if this stress response ramps up in space and time then recovery becomes increasingly difficult. First documented in 1998, global mass bleaching events have since occurred in 2010, 2014-17 and 2023-25 (Readfern, 2025). Contemplating reef change at such timespans makes for “an abrupt event in human time, an instantaneous event by any standards of evolution, let alone geology” (Veron, 2008, p. 206). The jump from distressed coral to bleached reefs, from localized event to global trend provides an entry-point for coral scientists and media actors chasing incontrovertible evidence of the scale of ongoing and accelerating global environmental change in the name of political consensus-building, conservation funding, and mass public influence (Moore, 2021). At the same time, such grave disturbances in the condition of coral scientists’ research object, in its availability to study and conservation, exert pressure upon their epistemic and evaluative practices (Jones, 2024).

Here are some human analogues to the cluster of symptoms that mass bleaching brings: fever, lethargy, brittle bones, and shortness of breath—acute and highly transmissible. But why even play at interspecies nosology? For one thing, such analogies work in the genre of Big Coral to connect the synchronized response of different coral species to the generalized hazard of ocean stress. Any one aerial survey image or before-and-after montage, for example, can depict many hundreds of kinds of coral, each a different biological species. But the register of health and illness has a rhetorical charge that reaches beyond the biology of individual corals. It has, moreover, been some time in the making. Sabina Leonelli and Rachel Ankeny (2020) have shown that, in recent decades, some coral scientists have strategically embraced an “infection repertoire” to characterize corals and their inquiry. They have used this repertoire to recast their object, secure resources, and extend their influence within international reef studies. The importance of researching the etiology of mass bleaching was key to this move. One early hypothesis, for example, ventured that bleaching had an adaptive purpose, allowing coral hosts to endure changing ocean conditions by breaking a harmful symbiotic relationship with one kind of algae so as to form a beneficial connection with another kind of algae present in the water column (Buddemeier & Fautin, 1993; Jones, 2008). Mass bleaching, according to this influential yet contested hypothesis, would be both a potentially fatal condition and an evolutionary opening. Thus, in mediating the biogeochemical dynamics of global ocean change, corals are not just living archives of the Earth’s “geohistory,” they now function as real-time indicators of “planetary health” (Farman & Rottenburg, 2019).^4^ The infection repertoire offers another way of appreciating why Big Coral can relay a sense of climatic disorder as at once happening and hypothetical, a phenomenon that oscillates between the treatable and the terminal. This confounding process recalls what Jain (2007, 2013) refers to as “living in prognosis,” a puzzling experience of present clarity over future calamity that—precisely because the bare fact of diagnosis is often devoid of ethical prescriptions—can feel like a confusing condition in need of treatment in its own right. One possibility of what this might look like is treating mass bleaching preventatively, so to speak, by accelerating corals’ predicted evolutionary trajectory. But what does this mean and how does this work? What visions of nature and the human does “human-assisted evolution” portend?

### Evolution interrupted and human-assisted

In the Winter of 2015, a Perspectives article in the *Proceedings of the National Academy of Sciences* suggested a new direction for coral science: “human-assisted evolution.” Borrowing from work in terrestrial ecology, the authors venture a hypothesis: if we can pre-emptively expose reef-building corals to warmer and more acidic conditions, then we might open up evolutionary pathways for their survival, growth, and reproduction beyond a predicted extinction cliff (van Oppen et al., 2015).

Assisted evolution—the “human-” qualifier is quickly dropped—draws on shifting biological understandings of ecological adaptation, natural selection and practices of animal husbandry. In keeping with the theory of natural selection, for a given species to endure beyond the conditions of its habituated environment, it must adapt to survive and reproduce with different resources. Some life forms appear to have a very broad latitude of habituation and continue their evolutionary journey uninterrupted even by mass extinction events—think crocodiles, horseshoe crabs, or gingko trees. Tracking how reef-building organisms respond to this same order of events requires shifting focus from the biological individual to the microbial community. Because, a leading explanation for “reef gaps” in the geological record is not the time required for individual reef-building organisms like corals to evolve. Rather, it is the time required for a compatible combination of host organism, symbiotic fungi, bacteria and photosynthesizing microalgae to evolve, link up, and sync up in space and time.^5^ This multiorganism assemblage is known as the coral “holobiont” and it powerfully articulates a collaborative theory of evolution grounded in the concept of “symbiosis,” which some environmental humanities scholars argue holds profound democratic potential for rethinking human–environment relations in the idiom of “symbiopolitics” (Gilbert et al., 2012; Haraway, 2016). For the purposes of assisted evolution as a bioscientific research program, however, symbiosis presents different avenues for adaptation that call for different experimental angles of approach (i.e., random genetic mutation or wholesale changes to corals’ chromosomal DNA, variations in gene expression or changes to what existing genes are replicated as RNA, community reorganization or changes to the composition of corals’ microbiome). Thus, assisted evolution anticipates a coming reef gap and proposes to avoid it by bringing into existence the kind of coral holobiont that would typically arise in its aftermath. As such, it can be understood as a practical attempt to embed the human within the coral holobiont in the name of companionate evolutionary time travel.

Assisted evolution is not a back of the envelope calculation. It is a carefully designed experimental program that blurs the lab/field divide, crosses the globe, and leans on the capabilities of a bespoke research instrument in Australia called the National Sea Simulator in order to breed, rear, and stress-test the coral holobiont in predicted-but-as-yet-non-existent ocean conditions. In under a decade, it has gone from experimental trial to research flagship at the Australian Institute of Marine Science, the federal science agency responsible for researching and advising on the nature of the Great Barrier Reef. From the outset, assisted evolution was an experimental program designed to test different possible ways of intervening in the evolutionary trajectory of reef-building corals. Its goal was to develop a conceptual and practical “toolbox” to use on a wide range of coral species thanks, in part, to parallel efforts in coral cryo-conservation.^6^ In this sense, assisted evolution not only draws on the genre of Big Coral to represent a new horizon of human-reef interactions, it also operationalizes Big Coral to make that horizon a reality.

Assisted evolution is one real-world example of a movement that has many names: ecomodernism, the new conservation science, restoration ecology, bio-, geo-, eco-, climate engineering, environmental interventionism, the Good Anthropocene (Asafu-Adjaye et al., 2015; Hobbs et al., 2006; Kareiva et al., 2007). It articulates a version of the following argument: the ongoing acceleration of climactic derangement shows that, despite overwhelming scientific consensus and decades of environmental organizing by state and non-state actors, the relationship between technological and civilizational progress is broken; to reset this relationship, technoscience must carve out a new frontier from within fundamental natural processes. From one aspect, this looks like abduction, a style of reasoning that draws conclusions from premises that lie just over a current horizon of reality in a manner that “joins hope to reason, present texts to future contexts, contemporary life forms to scientific forms of life yet to come” (Helmreich, 2009, p. 172). Yet, at the same time, this is hope laced with something else—a judgment that hovers between disappointment and disdain towards existing formations of politics for “inaction” on the problem of climatic derangement. The resulting diagnosis of runaway existential danger becomes the justification for trying out techniques long-considered “too risky” or “last resort” in the name of “enhancing” an ailing Earth. Examples include techniques of hybridization, de-extinction, rewilding, assisted migration, artificial refugia, genetic castration, and so on. This movement, like any other, has its ideologues, fair-weather friends, reluctant adherents, media relays, and unwitting conscripts whose commitments and ambivalences vary. Thus, some adherents see their work as bypassing the aforementioned political impasse while others imagine “buying time” for seemingly hesitant actors to gather their resolve. Either way, it suggests that the scale of the climate crisis provides a warrant to develop modalities of science and technology that explicitly break with existing moral and political conventions.

In recent years, humanistic and social scientific scholars have engaged and challenged this movement. Among other things, critics have underlined that the radical hope this movement conjures presumes an imperiously narrow political and moral imaginary that denies the longstanding entanglement of technoscience and politics.^7^ Here is a concise version of the broader charge: “Not content with the utopianism of modernity … the ecomodernists are also uchronists, as if they were living at a time when they alone were in command” (Latour, 2015, p. 224). I cite this work not to dwell on good and bad faith attempts to purify technoscience of politics but to raise a different question: “in command,” but of what? For utopianists, you would think that would be a place, the topos to come; for uchronists, then, a time, the chronos to come. For both, though, surely the stakes of being “in command” have to do with getting there? Using coral to get somewhere that does not yet exist is precisely what assisted evolution proposes by montaging one concept of change, assistance, with another, evolution. What does it mean to understand evolution as something to assist?

The researchers behind “assisted evolution” for reef-building corals borrowed the term from terrestrial ecology, specifically the work of United States Department of Agriculture ecologists Thomas Jones and Thomas Monaco. This duo contend that modified terrestrial ecosystems should not be returned to their historical composition in cases when the ecological niche required by previously endemic plant life has collapsed. Instead, ecologists should make use of a broader set of plant life and restoration techniques to bring such altered landscapes to the point of providing desired “ecological functions.” In their case study of the Great Basin, an expansive desert and shrubland region bounded by the Rocky Mountains and the Sierra Nevada, some examples of these functions include “controlling wildfire, inhibiting invasive plant populations, and restoring soil structure and nutrient dynamics” (2009, p. 542). To motivate their claims, they cite two major references in the “new conservation science” literature (i.e., Hobbs et al., 2006; Kareiva et al., 2007).

Assisted evolution for terrestrial ecosystems and coral reefs share the same goal of modifying the biological composition of disrupted ecologies to stabilize them as habitat. However the temporal, spatial, and biological calibration of each project is markedly different. The terrestrial ecologists argue for a pragmatic reckoning with permanently displaced plant life due to lost habitat in the name of introducing other lifeforms that can perform similar functions. The coral scientists argue for an anticipatory reckoning with foreseeably displaced marine life due to collapsing habitat in the name of making novel organisms that will avoid this process. There is a significant shift in scale from a local ecology to the global oceans, from the lost-for-good to the yet-to-be-salvaged. Scale, here, is not just a representational but a pragmatic category; it is doing work to stabilize the aims and practices of conservation biology in a context of generalized disturbance (Lempert & Carr, 2016; Hecht, 2018). Indeed, the scalar jump of assisted evolution is of a piece with Jones and Monaco’s argument, who all but invite such extension, writing:

“Our comments are based on this region as a case study and must not be construed as generally applicable to more malleable environments that are relatively responsive to restoration treatments; nevertheless, our remarks may apply to other, highly modified systems. In order to determine whether they apply to some other recalcitrant systems, we suggest posing the following questions: Are undesirable modifications reversible? Is it possible to restore species composition, ecological function, and successional and evolutionary processes that prevailed prior to disturbance?” (2009, p. 546)

*Our remarks may apply to other recalcitrant systems.* The “reef gap” seems eligible for Jones & Monaco’s test given the “undesirability” of a world without reef-building corals and their works. Applying this test, however, involves a temporal leap and a striking shift in scale: the global oceans become a single ecological niche undergoing uncertain evolution for which any and all coral-based restoration constitutes a desirable reversal. This transposition to foreseeably but not yet permanently displaced marine life holds against a Big Coral background. Moreover, it draws focus on something otherwise blurred in the terrestrial field, namely, a problem that has long been a feature of ecological thinking: the interdependency of the living and its milieu, the lab and field, and the question of where to locate the force that holds them together. To get a sense of this, consider that either of the following warrants would be grounds to endorse human-assisted evolution for corals per Jones & Monaco’s language: “the global oceans are recalcitrant, their modification due to Earth distress is undesirable and irreversible” or “corals are recalcitrant, their modification due to Earth distress is undesirable and irreversible.” The term I have substituted is not life or environment or ecology but “system.” The mutual constitution of ocean and coral as indices of damaged life appears to conform with systems thinking. At the same time, assisted evolution seems to repudiate such thinking by evidencing a desire to break the system the better to fix it, as it were, by experimentally reengineering it.

Yet at what timescale does a test like Jones and Monaco’s apply? It is axiomatic that the Earth is given to change and so, from an evolutionary perspective, there are no circumstances in which, for any given place, there won’t be some time at which natural selection operates to the detriment of the lifeforms that had been endemic to it up until that point. The “new conservation science” authors that the duo cite in support are quite up front about this, as well as the fact that people have profoundly shaped the nature of nature for millennia, stating “all ecosystems can be considered ‘novel’ when placed in the appropriate temporal context” (Hobbs et al., 2009, p. 599). They do, however, qualify the novelty within novelty:

Our current concern with novel ecosystems must thus be set in a longer timeframe, and questions of relative value compared with other ecosystem types should perhaps focus on the services either provided by or lost from particular types of ecosystem. It is, however, clear that rates of change are much faster in modern times and that, for better or for worse, new technologies help to overcome biogeographical and biophysical barriers to establishment (Hobbs et al., 2006, p. 3).

*For better or worse.* “New technologies” here function as a pharmakon, the cause of and treatment for one and the same calamity. More than this, though, they are invoked as an ahistorical entity, a timeless and inexorable presence whose development and operations are not to be understood as predicated upon actually existing human beings—who grow crops, drill wells, play games, patent compounds, publish articles, launch rockets, wage war, and so on—but as a disembodied “helping hand” for biogeophysics. Setting the new conservation biology within “a longer time-frame” resonates with the geoscientific coinage of the Anthropocene concept, wherein the so-called “geological agency of mankind” is not to be suffered but harnessed (Crutzen, 2002). It belies a faith in turning tragedy into triumph, as if to say: now that we know how we can break the Earth, we must know how we can repair it. In picturing the Earth as a biodynamic whole to be measured and corrected for through evaluations of “function” and “service,” an “evolved” technoscience imagines drawing a line under its prior structural complicities with the Earth’s transformation so as to confront a future of ever-accelerating ecological strains inviting specialized “assistance.” By doing so from within—reconceiving coral endangerment as an engine of renewal and not decline—assisted evolution challenges the presumed necessity of currently existing biology in the name of some other, yet to be determined agreement.

Let me close this section with two sets of summary points. First, there seem to be competing temporalities at work here: (1) assisted evolution is just natural selection, but accelerated, and so, as an aspect of an avowed symbiosis between humans and corals, coral engineering is timeless; (2) assisted evolution needs to be calibrated with societal expectations alongside similar developments in conservation biology and so coral engineering is continuous with human history; (3) assisted evolution is an unprecedented application of reparative technology that will alter the compact between science and society and so coral engineering is revolutionary. Second, even as assisted evolution leans upon the theory of natural selection, it also strains this theory’s operation by anticipating its bearing upon one life form—reef-building corals—with a view to interception and redirection. Yet is this just a glitch in the future course of coral evolution? Human beings, also, are a biological entity to whom natural selection applies. A symmetrical question therefore arises of whether acquiring the ability to “assist evolution” alters the evolutionary trajectory of the human.^8^ What does it mean to understand assisted evolution in these terms, not as laying claim to a new kind of coral but a new kind of human?

### Questioning super corals

The pace of technoscientific change presents conceptual and practical challenges for description: blink and you can miss it. But miss what? Within the human sciences, there is a strong tradition of questioning the modernist equivalence of technoscientific and civilizational progress as a just-so story that veils the way people put power and capital to work and get worked over by them in turn. Genealogy, in this sense, is not a method for investigating the inheritance of kinship ties (i.e., ancestry) or the transformation of biological functions (i.e., natural selection) but, rather, the reproduction of ideological commitments through patterned acts of human hostility and hospitality. One example would be an orientation towards nature as a resource to be converted into the means of economic exchange (Franklin, 2007; Rajan 2006). Another would be an orientation towards life as a condition of permanent exposure to danger (Ahuja, 2016; Evans & Reid, 2015). The point of such analyses is not to dispute the fact that the stuff of nature can be transformed into exchangeable goods or that living beings can experience harm. Rather, it is to observe that reducing experience to economic or biological existence, for example, locks political and moral reasoning into endless calculations of relative worth and vulnerability. Such calculations work through further abstractions—like utility, productivity, growth, efficiency, or resilience—that are concretized within institutional mandates, communal norms, and practices of self-fashioning. The foregoing genealogical sketch could be fruitfully extended in these directions.^9^ In this closing section, however, I will push further on the worry that assisted evolution seems to place over the reproducibility of life itself. My question has not been whether or not assisted evolution is something that some coral biologists can do but, instead, what to make of the fact it is something they can want to do and, moreover, can claim needs to be done. There is a puzzle not only in explaining assisted evolution but in pondering why such an explanation is necessary in the first place.^10^

Having identified whole scale intervention as a new horizon for conservation biology, the assisted evolution team quickly found themselves embroiled in standing disagreements over when and why to demarcate the study of biology from its manipulation. Researchers rebutted comparisons with genetically modified seedstock by emphasizing that their mode of intervention into coral genetics would remain indirect (at least at first); if there was an agricultural analogue to be had, they said, it would not be genetic modification but selective breeding. Many coral scientists continue to question the feasibility of direct intervention in the ecology of coral reefs (Hughes et al., 2023). The findings of assisted evolution are tentative and its authors cautious about their generalization (McLeod et al., 2022). Recent work, for example, emphasizes that a more viable engineering target may not be reef-building coral species per se but the multispecies microbial communities that regulate their lifeways (Chan et al., 2023). Strikingly, as this work develops, researchers have all but dropped the initial shorthand used to designate their quarry: “super corals.” One recent survey article, for example, cites “super-corals” as one contribution among many to a terminological debate over how to identify reef-building corals existing “at the edge of environmental limits” (Schoepf et al., 2023). Gone is the notion of evolutionary endings and escapism discussed above and in its place is a framework of “marginal” and “extreme” coral communities in different biogeochemical milieux. Rather than a world-historical conjuncture to confront, or a threat to the coevolution of humans and corals calling for biological speculation and speculative biologies, ocean stress becomes a spatially fragmented phenomenon materialized in the biology of different corals in global context. And so, instead of technobiological oracles, super corals become enfolded within the genre of Big Coral; the evolutionary anomaly becomes the generalized exception. Such shifting norms within research resonate with ongoing environmental changes: as marine heat waves continue to crest, the future ocean conditions once inputted in the National Sea Simulator are an increasingly present measure of reality.

I choose to dwell on the now seemingly outdated term “super corals” because it can help appreciate the strong claim upon the concept of evolution explained in the previous section and so suspend later normalization or equivocation. Despite hesitation among the leadership team, assisted evolution researchers offered “super corals” as a goad to science communication and it was successful as such. Ruth Gates, in one account, explains it by analogy:

“we rapidly increase the performance of talented athletes so they become super athletes by identifying them, training them in the gym, giving them great nutrition and then they often breed with people they meet in their training environment to produce extraordinarily talented offspring. This is exactly what we are doing with the work: identifying the best of the best”. (Braverman, 2018, p. 219)

*The best of the best.* In comparing natural selection to an athletic competition, the atmosphere of existential vulnerability that goes with either the Darwinian notion of the survival of the fittest or the terminal prospect of the reef gap recedes in favor of a benign and even engrossing picture of sporting prowess. Yet assisted evolution requires continuous performance, and thus the analogy becomes decidedly strange when Gates stretches it over time. The image of “super athletes” becoming broodstock, casually embracing eugenics in the name of transgenerational dominance throws up an ontological tremor between us and them, “we” who oversee the training regime and “they” who endlessly pursue the superlative imperative to be best.

It is difficult, then, to fully resolve the trouble that the “super” in super corals brings. And this might just be the point. Among other things, the term picks up on the distinction between the natural and the supernatural, an opposition crucial to a post-Enlightenment understanding of nature as disenchanted and available to explanation in strictly materialist terms. Anthropologist Abou Farman locates this distinction within a series of secular negations through which technoscience delimits the question of existence: “that the transcendent or supernatural can add nothing to proper explanations of reality, that the afterlife is illusory, that the universe has no goal or meaning, and that there are no spirits in things, or mind in nature” (Farman, 2020, p. 19). No matter how effective these negations are at stabilizing the authority of scientific descriptions of a disenchanted universe, he observes, they constantly highlight “gaps” in the human experience wherein fundamental questions of meaning, mind, and value go unanswered. Parascientific formations like medicine and the law scaffold their own secular authority on investing these gaps with social categories and operations, Farman argues. In death, for example, personhood is negotiated as the embodied human being transitions from patient to cadaver, living loved one to departed family member. How these gaps are bridged is not predetermined (think disputes over who counts as next of kin). That these gaps persist is a source of anxiety, perhaps especially in moments of conceptual breakdown. Indeed, Farman’s analysis emerges from an inquiry into the human life-extension movement also known as “transhumanism” whose momentum lies not only in offering a biological theory of life after death through, for example, cryopreservation, but in conceptually and practically challenging the standing legal, medical, and cultural regime surrounding the transition from life to death. Time and again, this alternative “immortalist regime” enfolds the technoscientific claims of life-extension with a more-than-secular myth of posthuman flourishing for all pursued by a select few.

Part of the rhetorical force of “super corals,” then, lies in ostentatiously overstepping the secular norms of technoscience, which assume that metaphysical puzzlement about the nature of nature can be bracketed while writing up an impersonal and disembodied description of the material universe. It is in this sense—and not only by analogy with debates over genetically modified seedstock—that we can appreciate why assisted evolution researchers repeatedly found themselves defending that they were “not playing God,” whether by underscoring the long history of human encroachments upon coral reefs or the essential timelessness of evolution. As Ruth Gates put it in one interview: “we’re doing what nature has done all the time, but can’t do quickly enough” (Braverman, 2018, p. 243). Yet therein lies the trouble, and also the play: taking a description of fact, the gradual evolution of corals and the continuous impact of humans on their milieu, as the basis for more-than-natural intervention, the targeted acceleration of said evolution and impact.^11^ Indeed, recall that it is precisely the continuous operation of the “law” of natural selection that assisted evolution seeks to suspend in its attempt to avoid coral terminality and the predicted reef gap. The biosciences decide the exception to the operation of the very laws of nature upon which they stake their authority, the better to defend the continuity of these same laws while climate politics catches up. As an expression of frustration at the slow pace of evolutionary change and political progress, such a reaction may be all too human.^12^ Yet, strangely, it also calls for new powers of the human that combine the awesome reach of geological theory, and its backwards glance over hundreds of millions of years of evolution, with the practice of the experimental biosciences, and its promise of accelerating evolutionary time in the blink of an eye. (Recall the earlier gloss on mass coral bleaching: “an abrupt event in human time, an instantaneous event by any standards of evolution, let alone geology.”) Exercising these powers presumes that “we” are no longer the kinds of beings to whom evolution applies but must become the kinds of beings who apply evolution.

Let’s return, then, to Latour’s remark: “not content with the utopianism of modernity … the ecomoderns are also uchronists, as if they were living in a time in which they alone were in command.” *As if they were living.* It is possible to read Latour’s remarks as moral judgment, indicting the aspiring ecomodernists in his midst. It is also possible, in light of the above, to read them as ethnographic description. The uchronist is a flickering subject, who seems to wink in and out of existence, unmoored from time and space and no longer obviously subject to the laws of nature but revising them. Assisted evolution occupies a temporal horizon that is outside of history yet capable of reordering it, not unlike messianic time. Two decades ago, philosopher Mary Midgley waded into debate over the engineering of life and worlds and sought to clarify the strength of resistance encountered from lay observers. Her point was not to articulate a decisive argument for or against such activities but rather to invite greater appreciation for the moral trouble they occasion.

Midgley develops three explanations in a section from *The Myths We Live By* (2003) tellingly titled “The Supernatural Engineer.” First, biological claims upon the genesis and development of living entities do not simply unsettle the reproductive futures of specific lifeforms. They lay claim to nature in a generic sense by way of more open-ended concepts, such as species and evolution. Second, the fact that a very small group of people should take it upon themselves to do so only adds to the trouble. After all, people (including you and I but also, no less, someone who happens to work at bioengineering) inherit and make do with concepts like "species" and "evolution" as we go about our ordinary lives. Hence, as mentioned earlier, why some environmental humanities scholars discern politico-moral force in findings that biological evolution is symbiotic all the way down. And third, obsessing over whether or not such powers of intervention are right or wrong, legitimate or illegitimate, governed or ungoverned, can become a distraction from confronting the broader difficulty behind the living problems to which they claim to respond. It is easier, Midgley suggests, to debate the merits of transgenic plants than to confront systemic failures within global food distribution (see also Borghini et al., 2020). To rephrase: it is easier to debate who or what form predicted-but-as-yet-non-existent human powers should take than to confront what is wrong with the current ones. Innovation, for Midgley, is a tempting but ultimately deceptive substitute for confrontation because it commits people to a dreamland purified of aggression, violence, destructiveness and other aspects of the lives of the animals we are, aspects that may be regrettable but that bear upon why we continue to commit our creative powers to such destructive ends: “In order to dig out something so deep in our psyches,” she writes “we do indeed need to reverse it explicitly in practice. The painful words WE WERE WRONG must not only be spoken but spelt out in action, and this needs to be action with a strong symbolism that bears on the offences that have been central to our crimes” (Midgley, 2003, p. 175).

## Conclusion: belated dreaming

This article on the time-traveling fantasy of Big Coral and assisted evolution has sought to understand the conceptual trouble occasioned when technoscience harnesses one charismatic “planetary” lifeform in a bid to change global environmental change. I have argued that the engineering of marine life and worlds in response to ocean stress bundles together evaluations of nature with evolutionary theorizing. I offered the genre of Big Coral and a genealogical sketch of assisted evolution to show how biogeochemical descriptions combine with affective and practical reasoning in ways that load up climate action with pressing philosophical and moral questions that exceed the “climate problem” per se. To conclude, I would like to return to my opening remark that there is something belated to this inquiry because, on the one hand, the movement to salvage the whole Earth has built up a head of steam, and because, on the other, we might not need new knowledge of global environmental change.

It is sometimes said that we live in a time of “late liberalism” or “late capitalism” or “late modernity” or “late industrialism,” expressions which hover between the idea that now is the last stage of these formations and the idea that the fantasies of the good life these formations promised never arrive on time for most people (Berlant, 2011; Povinelli, 2011, pp. 25–29). Meanwhile, a number of recent texts written in the shadow of planetary endings propose a confrontation with finitude that emphasizes the transformative power of humility (Scranton, 2015), mourning (Lear, 2022), or finiteness (Marder, 2023). And so, this article is belated in a simpler sense: it is a response to a response to ocean stress, a commentary on some of the presuppositions, avowals, and rationalizations accompanying a new kind of action: the intentional and open-ended embedding of human doings within coral evolution. It might be worth asking, then, whether assisted evolution could foster what Donna Haraway (2016) calls “response-ability”: an explicit posture of attention towards how human beings “become with” their more-than-human others. From one aspect, the question seems counterintuitive, given the aura of human exceptionalism that clings to interventionism itself, which jars with the ontological egalitarianism of Haraway’s writings. However, from another aspect and in a less triumphant key, the question invites another: why assume that “becoming with” is creative rather than destructive, democratic rather than imperious?^13^

Philosopher Stanley Cavell takes a different approach to the place of responsiveness within the human condition. He invites us to consider not whether we are able to respond to one another but the direction we emphasize as we do. In discussing typical approaches to the problem of knowledge, which for him means what we might be aiming at doing when we claim to know something, he says that people often seem to understand their ability to respond as the act of going in one particular direction—from self to other, near to far, here to there, known to unknown. This, he says, is a mistake, albeit an all too understandable one. It is, as it were, less a response than a reaction to the fact that the lives of the animals we are involves, first and foremost, exposure to the world, which goes before us and carries on after us, whose unknowns outpace our own claims to know, which are but ways into the separateness proper to the human condition. The problem of this separateness can be figured in the desire to span a coming “reef gap” by evolving corals to jump over it or, more generally, in the relentless identification of “knowledge gaps” for research to fill and so change climate change. This separateness takes quieter forms too, as in the disconnect, to paraphrase Alfred North Whitehead, between the red glow of a sunset and the particles and waves that make it so. As I understand it, Cavell’s point is not to venerate separateness but to acknowledge it as the tentative condition under which, nevertheless, we inherit and transform an ability to think, feel, and act.

We might, then, ask: what does it mean for some human beings to claim responsibility for the ability of corals to respond to climatic derangement? For one, such a claim is a challenge to the separation of human from coral life, because of the metonymy it threads between reef-building corals as living entities and their condition as existential threat detectors. It asserts a picture of the coral and the human as bound together in some higher order experiment with biological enhancement. In scholarship discussing a speculative future such as this, an all but obligatory point of passage is Philip K. Dick’s 1968 science fiction *Do Androids Dream of Electric Sheep?* The novel tells of a world ravaged by environmental destruction, corporate greed, and collective inaction in which, at one point in time, an engineer designed an android worker capable of enduring harsh “off world” conditions thereby supplying the labor needed to sustain, in some form, mass society and the global order. These androids are known as “replicants” and their resemblance to real humans eventually became so sophisticated that it took special agents known as “blade runners” to tell them apart from humans. The need for blade runners is a security state response to the way that replicants who learned of their engineered nature display a tendency to go “rogue,” particularly in response to the fact that their lifespan is preset and preemptively shortened in a gesture of built-in, managed obsolescence. The novel centers around one group of rogue replicants who return to Earth to confront their maker for an evolutionary upgrade and the blade runner who is tasked with their termination, Rick Deckard.

*Do Androids?* is often understood to hang on the question of whether or not Deckard is a replicant (and, by transitivity, whether or not Deckard the character is the fictional double of Dick the author). Yet anthropologist Deborah Battaglia (2001) has written that it is not the answer that matters but the question itself, for it highlights that there is something disturbing to the existence of doubles or clones or copies or replicants that do not obviously reveal themselves as such. Put differently, the ontological ambivalence of the double scrambles our standing repertoire of responses to otherness. Battaglia’s point offers a way into a less frequently discussed dimension of the novel, that the technology of replication is not just applied to humans but to a panoply of nonhuman animals who, in an act of moral rescue for a natural world devastated by extinction, are also subject to replication and become coveted as beloved companions and status trophies. The same question of ontological integrity hovers over these beings, whose authenticity is greatly valued and conveys enormous prestige upon their “owners” given the perennial possibility of, unwittingly, mistaking a copy for the real thing. The mark of that mistake, however, is not a matter of knowledge but of sensibility. Thought experiments about animal suffering are, for example, a mainstay of the cognitive tests that blade runners use to tell replicant from human. The blade runner Deckard, meanwhile, reads as his most human precisely in extended passages when, whether in waking life or in dreams, like a child learning to play with concepts and their edges, he pursues the company of a likely-but-as-yet-unconfirmed replicant frog.

It is possible to acknowledge the death-defying confidence trick of the engineering corporation who unleashed an interstitial form of life that weaves together human, nonhuman, and machinic becoming. Yet just like the replicant production line, the trick itself is never one and done, but serialized and biologically contagious. The corporation’s Promethean push to perfect replication, to sustain the reproduction of one social order by developing another in parallel, goes hand in hand with the possibility of losing the question of whether Deckard is a replicant or not, and who he would become without it. Do corals dream of simulated seas? Some of the time, some of us, human beings, do. What is at stake in this question is less the efficacy of an experimental technique but rather what such dreams are for, whether salvaging a symbiotic yet fragile connection between humans and corals or wishing it away. Either way, we might do well to remember that dreams can, sometimes, take on a life of their own.

## Data Availability

The authors confirm that the data supporting the findings of this study are available within the article and its supplementary materials.

## Footnotes

1 For a discussion of the concept of “existential” or “catastrophic” risk, see Edwards (2024) The enthusiasm for making the Earth’s biogeochemistry available to engineering rides on the serial commissioning, coordination, outreach, and circulation of reports from national governments and supranational institutions (IPCC, 2018; Lawrence and Schäfer, 2019; NASEM, 2019; Shepherd, 2012). This present-day momentum, however, should be understood in relation to geopolitical and geoscientific shifts after World War II (Fleming, 2010; Schaefer, 1968). Meanwhile, a world away from the deliberations of supranational institutions, the pull of whole Earth salvage—and its attendant presumption of human mastery over nature through technoscientific advancement—takes more ordinary registers, as well, as in the following description of a first-year course required for a Bachelor of Science degree at an Australian university: “The ambition to shape our habitat and to understand the universe in which we reside has occurred in all cultures and across all epochs. The human need to know more and to do more has led to the rapid expansion of knowledge and technology that we see around us and is largely driven by advances in science. In this subject, we adopt an interdisciplinary approach to investigate how today’s science might be harnessed to make tomorrow’s world a better place, by addressing the most significant challenges that seem likely to emerge in our future: climate change and environmental sustainability” (The University of Melbourne, n.d.).

2 For an important discussion of Agamben’s concepts and the pressure that contemporary conceptualizations of marine molecular biology place upon them, see Helmreich (2009, pp. 100–105).

3 Hecht (2018) offers a powerful example of how to think with and against the universalizing scale of the Anthropocene, by following scale-jumping entities so as to emplot and emplace political imagination.

4 Another dimension of the medicalization of coral science is growing concern over the nature and spread of poorly-understood infectious diseases under conditions of rapid ocean change (Harvell et al., 2007).

5 Exactly how this happens is the subject of ongoing disagreement within the coral science community. One recent paper notes that some species of coral have persisted across mass extinction events, albeit not as reef-builders (Dishon et al., 2020).

6 The four initial experimental tracks were (1) “transgenerational” thermal stress testing to prompt genetic and epigenetic adaptation; (2) direct manipulation of coral microbiome, aka “probiotics for corals”; 3) crossbreeding of coral species to test for so-called “hybrid vigor”; (4) thermal stress testing and chemical mutation of zooxanthellae to then be injected into corals.

7 Two representative short examples are Hamilton (2016) and Szerszynski (2016). It is also instructive to read Latour’s (2011, 2015) initial equivocation and subsequent renunciation in *The Breakthrough Journal*. Eileen Crist has also been a vocal critic of the movement, and especially effective at rallying attention within the conservation milieu proper (Crist, 2014; Wuerthner et al., 2015). The debate “about” these speculative frontiers resonates with debates “about” the concept of the Anthropocene (Bonneuil & Fressoz, 2016). In noting this, it is possible to imagine that the antagonizing rhetoric of a document like “The Ecomodernist Manifesto” was designed to provoke learned debate in order to legitimize the very idea of an abstract, decontextualized, whole Earth “nature-as-usual” from which to break. For a fuller discussion of such circuits of enjoinment and enjoyment, see Mazzarella et al. (2019).

8 To say nothing of the fact that natural selection has its own intellectual history within the dominant tradition of biology whose status may be hegemonic yet is no metaphysical given.

9 On the question of ecological restoration as an on-ramp to capital accumulation, for example, see Huff and Brock (2023).

10 I am borrowing a move from Michael Taussig, who for decades has been developing a way of writing the weave of violence and healing proper to globalized modernity, which he refers to as the “nervous system.” The latter is messy, mobile, and wildly human in a way that defies the compulsory rationality of what he calls “agribusiness writing” to name the discursive backcloth to real-world practices of capitalist extraction and institutional disenchantment. Thus, “agribusiness writing assumes the need for an explanation when what is at issue is why one is required, and what is an explanation and how do you do one, and how weird is that?” (Taussig 2015, p. 5).

11 If nothing else, such a claim takes liberties with a strictly materialist philosophical position, wherein being is conditioned by the physical properties of the universe, by explicitly anticipating becoming. While I cannot elaborate upon this remark here, one point of departure is the pressure that moral thought places upon philosophical materialism and dualism as discussed in Crary (2016, pp. 38–91).

12 “The secular immortality regime culturally eliminates the possibility of an afterlife, thereby rendering final the end of the person at a certain point in the trajectory of the body. Yet secular time continues, and more important, it continues not as repetition or the eternal return of the same but via the secular notion of progress, a future ever changing, ever filled with the promise of better things. Suspension is the secular space in which the promise of the unfolding future can assume material embodiment and in scientific garb negate the absurdities of individual death” (Farman 2020, p. 40)*.*

13 It bears noting that Haraway has long centered non-innocence in her accounts of the entanglement of science and society.

## References

[CR1] Asafu-Adjaye, J., Blomquist, L., Brand, S., Brook, B. W., DeFries, R., Ellis, E., Foreman, C., Keith, D., Lewis, M., & Lynas, M. (2015) An ecomodernist manifesto.

[CR2] Adler, A. (2019). *Neptune’s laboratory: Fantasy, fear, and science at sea*. Harvard University Press.

[CR3] Ahuja, N. (2016). *Bioinsecurities: Disease interventions, empire, and the government of species*. Duke University Press.

[CR4] Ankeny, R. A., & Leonelli, S. (2020). Using repertoires to explore changing practices in recent coral research. In K. Matlin & J. Maienschein (Eds.), *Why study biology by the sea* (pp. 249–270). University of Chicago Press.

[CR5] Battaglia, D. (2001). Multiplicities: An anthropologist’s thoughts on replicants and clones in popular film. *Critical Inquiry,**27*(3), 493–514. 10.1086/449018

[CR6] Berlant, L. (2007). On the case. *Critical Inquiry,**33*(4), 663–672. 10.1086/521564

[CR7] Berlant, L. (2011). *Cruel optimism*. Duke University Press.

[CR8] Bonneuil, C., & Fressoz, J.-B. (2016). *The shock of the anthropocene: The earth, history, and us*. Verso.

[CR9] Borghini, A., Piras, N., & Serini, B. (2020). Ontological frameworks for food utopias. *Rivista di Estetica,**75*, 120–142. 10.4000/estetica.7375

[CR10] Braverman, I. (2018). *Coral whisperers: Scientists on the brink*. University of California Press.

[CR11] Bredekamp, H. (2008). *Les coraux de Darwin: Premiers modèles de l’évolution et tradition de l’histoire naturelle*. Translated by Christian Joschke. Les presses du réel.

[CR12] Bright, D. (2023). Coral. In J. Jacobs & A. Malinowska (Eds.), *Microbium: The neglected lives of micromatter* (pp. 47–64). Punctum Books.

[CR13] Buddemeier, R. W., & Fautin, D. G. (1993). Coral bleaching as an adaptive mechanism. *BioScience,**43*(5), 320–326.

[CR14] Chakrabarty, D. (2009). The climate of history: Four theses. *Critical Inquiry,**35*(2), 197–222. 10.1086/596640

[CR15] Chakrabarty, D. (2019). The planet: An emergent humanist category. *Critical Inquiry,**46*(1), 1–31. 10.1086/705298

[CR16] Chan, W. Y., Meyers, L., Rudd, D., Topa, S. H., & van Oppen, M. J. H. (2023). Heat-evolved algal symbionts enhance bleaching tolerance of adult corals without trade-off against growth. *Global Change Biology,**29*(24), 6945–6968. 10.1111/gcb.16987 37913765 10.1111/gcb.16987

[CR17] Collingwood, R. G. (1960). *The idea of nature*. Oxford University Press.

[CR18] Colman, F. (2023). Quantum feminicity: Modes of countermanding time. *Technophany,**2*(1), 1–37. 10.54195/technophany.14048

[CR19] Crary, A. (2016). *Inside ethics*. Harvard University Press.

[CR20] Crist, E. (2014). Ptolemaic environmentalism. In G. Wuerthner, E. Crist, & T. Butler (Eds.), *Keeping the wild* (pp. 16–30). Island Press. 10.5822/978-1-61091-559-5_3

[CR21] Crutzen, P. (2002). Geology of mankind. *Nature,**415*(6867), 23–23. 10.1038/415023a 11780095 10.1038/415023a

[CR22] Dishon, G., Grossowicz, M., Krom, M., Guy, G., Gruber, D. F., & Tchernov, D. (2020). Evolutionary traits that enable scleractinian corals to survive mass extinction events. *Scientific Reports,**10*(March), 3903. 10.1038/s41598-020-60605-2 32127555 10.1038/s41598-020-60605-2 PMC7054358

[CR23] Edwards, P. N. (2024). Is climate change ungovernable? *London Review of International Law*. 10.1093/lril/lrae017

[CR24] Elias, A. (2019). *Coral empire: Underwater oceans, colonial tropics, visual modernity*. Duke University Press.

[CR25] Evans, B., & Reid, J. (2015). *Resilient life: The art of living dangerously*. Polity.

[CR26] Farman, A. (2020). *On not dying: Secular immortality in the age of technoscience*. University of Minnesota Press.

[CR27] Farman, A., & Rottenburg, R. (2019). Measures of future health, from the nonhuman to the planetary. *Medicine Anthropology Theory,**6*(3), 1–28.

[CR28] Fleming, J. (2010). *Fixing the sky. The checkered history of weather and climate control*. Columbia University Press.

[CR29] Franklin, S. (2007). *Dolly mixtures: The remaking of genealogy*. Duke University Press.

[CR30] Gates, R. D., Baghdasarian, G., & Muscatine, L. (1992). Temperature stress causes host cell detachment in symbiotic cnidarians: Implications for coral bleaching. *The Biological Bulletin,**182*(3), 324–332. 10.2307/1542252 29304594 10.2307/1542252

[CR31] Gilbert, S. F., Sapp, J., & Tauber, A. I. (2012). A symbiotic view of life: We have never been individuals. *The Quarterly Review of Biology,**87*(4), 325–341. 23397797 10.1086/668166

[CR32] Hadot, P. (2006). *The veil of Isis: An essay on the history of the idea of nature. *Translated by Michael Chase. Harvard University Press.

[CR33] Hamilton, C. (2016). The theodicy of the ‘Good Anthropocene.’ *Environmental Humanities,**7*(1), 233–238.

[CR34] Haraway, D. (1989). *Primate visions: Gender, race, and nature in the world of modern science*. Routledge.

[CR35] Haraway, D. (2016). *Staying with the trouble: Making kin in the chthulucene*. Duke University Press.

[CR36] Harvell, D., Jordán-Dahlgren, E., Merkel, S., Rosenberg, E., Raymundo, L., Smith, G., Weil, E., & Willis, B. (2007). Coral disease, environmental drivers, and the balance between coral and microbial associates. *Oceanography,**20*, 172–195.

[CR37] Hecht, G. (2018). Interscalar vehicles for an African Anthropocene: On waste, temporality, and violence. *Cultural Anthropology,**33*(1), 109–141.

[CR38] Heise, U. K. (2016). *Imagining extinction: The cultural meanings of endangered species*. University of Chicago Press.

[CR39] Helmreich, S. (2016). How like a reef: Figuring coral 1839–2010. In *Sounding the limits of life* (pp. 48–61). Princeton University Press.

[CR40] Helmreich, S. (2009). *Alien ocean: Anthropological voyages in microbial seas*. University of California Press.

[CR41] Helmreich, S. (2023). *A book of waves*. Duke University Press.

[CR42] Helmreich, S., & Roosth, S. (2010). Life forms: A keyword entry. *Representations,**112*(1), 27–53. 10.1525/rep.2010.112.1.27

[CR43] Hobbs, R. J., Arico, S., Aronson, J., Baron, J. S., Bridgewater, P., Cramer, V. A., Epstein, P. R., Ewel, J. J., Klink, C. A., & Lugo, A. E. (2006). Novel ecosystems: Theoretical and management aspects of the new ecological world order. *Global Ecology and Biogeography,**15*(1), 1–7.

[CR44] Hobbs, R. J., Higgs, E., & Harris, J. A. (2009). Novel ecosystems: Implications for conservation and restoration. *Trends in Ecology & Evolution,**24*(11), 599–605. 10.1016/j.tree.2009.05.012 19683830 10.1016/j.tree.2009.05.012

[CR45] Hoegh-Guldberg, O. (1999). Climate change, coral bleaching and the future of the world’s coral reefs. *Marine and Freshwater Research,**50*(8), 839–866.

[CR46] Hu, C. (2024). Postcolonial technoscience revisited. *Social Studies of Science*. 10.1177/03063127241303778 10.1177/03063127241303778 39686535

[CR47] Hu, C. (2025). Fracking and historicizing: On deepened time in west Texas. *Cultural Anthropology,**40*(2), 354–383.

[CR48] Huff, A., & Brock, A. (2023). Introduction: Accumulation by restoration and political ecologies of repair. *Environment and Planning E: Nature and Space,**6*(4), 2113–2133. 10.1177/25148486231168393

[CR49] Hughes, T. P., Baird, A. H., Morrison, T. H., & Torda, G. (2023). Principles for coral reef restoration in the Anthropocene. *One Earth,**6*(6), 656–665. 10.1016/j.oneear.2023.04.008

[CR50] Hughes, T. P., Barnes, M. L., Bellwood, D. R., Cinner, J. E., Cumming, G. S., Jackson, J. B. C., Kleypas, J., Van De Leemput, I. A., Lough, J. M., & Morrison, T. H. (2017). Coral reefs in the Anthropocene. *Nature,**546*(7656), 82–90. 28569801 10.1038/nature22901

[CR51] IPCC (2018). Summary for policymakers. In *Global warming of 1.5° C. An IPCC special report on the impacts of global warming of 1.5° C above pre-industrial levels and related global greenhouse gas emission pathways, in the context of strengthening the global response to the threat of climate change* (Vol. 32). Intergovernmental Panel on Climate Change.

[CR52] Jacobsen, R. (2016, October 11). Obituary: Great Barrier Reef (25 Million BC–2016). *Outside Magazine*. https://www.outsideonline.com/2112086/obituary-great-barrier-reef-25-million-bc-2016.

[CR53] Jain, L. (2007). Living in prognosis: Toward an elegiac politics. *Representations,**98*(1), 77–92. 10.1525/rep.2007.98.1.77

[CR54] Jain, L. (2013). *Malignant: How cancer becomes us*. University of California Press.

[CR55] Jones, E. (2024). Exploring the socio-ecology of science: The case of coral reefs. *European Journal for Philosophy of Science,**14*(3), 29. 10.1007/s13194-024-00589-2

[CR56] Jones, R. J. (2008). Coral bleaching, bleaching-induced mortality, and the adaptive significance of the bleaching response. *Marine Biology,**154*(1), 65–80. 10.1007/s00227-007-0900-0

[CR57] Jones, T. A., & Monaco, T. A. (2009). A role for assisted evolution in designing native plant materials for domesticated landscapes. *Frontiers in Ecology and the Environment,**7*(10), 541–547.

[CR58] Kareiva, P., Watts, S., McDonald, R., & Boucher, T. (2007). Domesticated nature: Shaping landscapes and ecosystems for human welfare. *Science,**316*(5833), 1866–1869. 10.1126/science.1140170 17600209 10.1126/science.1140170

[CR59] Klein, J. (2017, August 17). No limits. *Platypus, The CASTAC Blog* (blog). https://blog.castac.org/2017/08/no-limits/.

[CR60] Kolbert, E. (2021). *Under a white sky: The nature of the future*. Crown.

[CR61] Lakoff, A., & Keck, F. (2013). Preface: Sentinel devices. *Limn*, *1*(3), 2–3.

[CR62] Latour, B. (2011). Love your monsters. *Breakthrough Journal,**2*(11), 21–28.

[CR63] Latour, B. (2015). Fifty shades of green. *Environmental Humanities,**7*(1), 219–225.

[CR64] Lawrence, M. G., & Schäfer, S. (2019). Promises and perils of the Paris agreement. *Science,**364*(6443), 829–830. 10.1126/science.aaw4602 31147507 10.1126/science.aaw4602

[CR65] Lear, J. (2022). *Imagining the end: Mourning and ethical life*. Harvard University Press.

[CR66] Lempert, M., & Summerson Carr, E. (2016). *Scale: Discourse and dimensions of social life*. University of California Press.

[CR67] Lough, J. M., & Cooper, T. F. (2011). New insights from coral growth band studies in an era of rapid environmental change. *Earth-Science Reviews,**108*(3), 170–184. 10.1016/j.earscirev.2011.07.001

[CR68] Marder, M. (2023). *The phoenix complex: A philosophy of nature*. MIT Press.

[CR69] Marx, K. (1981). *Capital I: A critique of political economy*. Penguin Books.

[CR70] Masco, J. (2017). The crisis in crisis. *Current Anthropology,**58*(S15), S65–S76.

[CR71] Masco, J. (2020a). Anticipation. In C. Howe & A. Pandian (Eds.), *Anthropocene unseen: A lexicon* (pp. 35–38). Punctum Books.

[CR72] Masco, J. (2020b). *The future of fallout, and other episodes in radioactive world-making*. Duke University Press.

[CR73] Mazzarella, W., Santner, E. L., & Schuster, A. (2019). *Sovereignty Inc: Three inquiries in politics and enjoyment*. University of Chicago Press.

[CR74] McCalman, I. (2014). *The reef: A passionate history*. Farrar, Straus and Giroux.

[CR75] McCalman, I. (2017). Linking the local and the global. What today’s environmental humanities movement can learn from their predecessor’s successful leadership of the 1965–1975 war to save the great barrier reef. *Humanities,**6*(4), 77.

[CR76] McKean, C. A. (2024). Temporal volumes and dimensional friction in a coral core laboratory. *Time & Society,**33*(3), 251–282. 10.1177/0961463X241260772

[CR77] McLeod, I. M., Hein, M. Y., Babcock, R., Bay, L., Bourne, D. G., Cook, N., Doropoulos, C., Gibbs, M., Harrison, P., Lockie, S., & van Oppen, M. J. (2022). Coral restoration and adaptation in Australia: The first five years. *PLoS ONE,**17*(11), e0273325. 10.1371/journal.pone.0273325 36449458 10.1371/journal.pone.0273325 PMC9710771

[CR78] Merchant, C. (2003). *Reinventing Eden: The fate of nature in western culture*. Routledge.

[CR79] Midgley, M. (2003). *The myths we live by*. Routledge.

[CR80] Moore, A. (2021). We’ve never seen anything like it: Witnessing coral death and resurrection. *HAU Journal of Ethnographic Theory,**11*(2), 461–474. 10.1086/716237

[CR81] Moore, J. (Ed.). (2016). *Anthropocene or capitalocene: Nature, history, and the crisis of capitalism*. PM Press.

[CR82] Morton, T. (2018). *Being ecological*. Penguin.

[CR84] NASEM. (2019). *A decision framework for interventions to increase the persistence and resilience of coral reefs*. National Academy of Sciences, Engineering and Medicine.

[CR85] Phillips, A. (2000). An answer to questions. In *Promises, promises* (pp. 174–80). Faber and Faber.

[CR86] Povinelli, E. A. (2011). Economies of abandonment. Social belonging and endurance in late liberalism. In *Economies of abandonment*. Duke University Press.

[CR87] Rajan, K. S. (2006). *Biocapital: The constitution of postgenomic life*. Duke University Press.

[CR120] Readfern, G. (2025). More than 80% of the world’s reefs hit by bleaching after worst global event on record. *The Guardian,* April 23, 2025. https://www.theguardian.com/environment/2025/apr/23/coral-reef-bleaching-worst-global-event-on-record

[CR101] Rivera-Sotelo, A-S. (2024). Elusive coral and fish: Reconsidering the shore-offshore separation in Caribbean archipelagos. *The Journal of Latin American and Carribean Anthropology,**29*(1), 5–16.

[CR88] Rosol, C., Schäfer, G. N., Turner, S. D., Waters, C. N., Head, M. J., Zalasiewicz, J., Rossée, C., Renn, J., Klingan, K., & Scherer, B. M. (2023). Evidence and experiment: Curating contexts of Anthropocene geology. *The Anthropocene Review,**10*(1), 330–339. 10.1177/20530196231165621

[CR89] Rozwadowski, H. M. (2009). *Fathoming the ocean: The discovery and exploration of the deep sea*. Harvard University Press.

[CR90] Rudwick, M. (2014). *Earth’s deep history: How it was discovered and why it matters*. University of Chicago Press.

[CR91] Ruetsche, L. (2004). Virtue and contingent history: Possibilities for feminist epistemology. *Hypatia,**19*(1), 73–101.

[CR92] Schaefer, V. (1968). The early history of weather modification. *Bulletin of the American Meteorological Society,**49*(4), 337–342.

[CR93] Schäfer, S. (2023). The intervention of climate science. In Z. Baker, T. Law, M. Vardy, & S. Zehr (Eds.), *Climate, science and society* (pp. 261–267). Routledge.

[CR94] Schoepf, V., Baumann, J. H., Barshis, D. J., Browne, N. K., Camp, E. F., Comeau, S., Cornwall, C. E., Guzmán, H. M., Riegl, B., Rodolfo-Metalpa, R., & Sommer, B. (2023). Corals at the edge of environmental limits: A new conceptual framework to re-define marginal and extreme coral communities. *Science of the Total Environment,**884*, 163688. 10.1016/j.scitotenv.2023.163688 37105476 10.1016/j.scitotenv.2023.163688

[CR95] Schuster, J. (2020). Coral cultures in the Anthropocene. *Cultural Studies Review,**25*(1), 85–102. 10.3316/informit.730939900867595

[CR96] Scott, D. (2011). The technological fix criticisms and the agricultural biotechnology debate. *Journal of Agricultural and Environmental Ethics,**24*(3), 207–226. 10.1007/s10806-010-9253-7

[CR97] Scranton, R. (2015). *Learning to die in the Anthropocene: Reflections on the end of a civilization*. City Lights Publishers.

[CR98] Sennett, R. (2008). *The craftsman*. Yale University Press.

[CR99] Shepherd, J. G. (2012). Geoengineering the climate: An overview and update. *Philosophical Transactions of the Royal Society A: Mathematical, Physical and Engineering Sciences,**370*(1974), 4166–4175. 10.1098/rsta.2012.0186 10.1098/rsta.2012.0186 22869795

[CR100] Slezak, M. (2016, June 6). The Great Barrier Reef: A catastrophe laid bare. *The Guardian*. https://www.theguardian.com/environment/2016/jun/07/the-great-barrier-reef-a-catastrophe-laid-bare

[CR102] Sponsel, A. (2018). *Darwin’s evolving identity: Adventure, ambition, and the sin of speculation*. University of Chicago Press.

[CR103] Sponsel, A., Gillis, J., & Torma, F. (2015). *From Cook to Cousteau: The many lives of coral reefs*. White Horse Press.

[CR104] Stoddart, D. (2001). Be of good cheer, my weary readers, for I have espied land. *Atoll Research Bulletin,**494*(12), 235–272.

[CR105] Szerszynski, B. (2016). Getting hitched and unhitched with the ecomodernists. *Environmental Humanities,**7*(1), 239–244. 10.1215/22011919-3616443

[CR106] Taussig, M. (2015). *The corn wolf*. University of Chicago Press.

[CR83] The University of Melbourne. (n.d. ). Today’s science, tomorrow’s world (SCIE10005). In *The University of Melbourne handbook*. Accessed November 27, 2023. https://handbook.unimelb.edu.au/2022/subjects/scie10005.

[CR107] van Oppen, M. J. H., Oliver, J. K., Putnam, H. M., & Gates, R. D. (2015). Building coral reef resilience through assisted evolution. *Proceedings of the National Academy of Sciences,**112*(8), 2307–2313. 10.1073/pnas.1422301112 10.1073/pnas.1422301112 PMC4345611 25646461

[CR108] Veron, J. E. N. (2008). *A reef in time: The Great Barrier Reef from beginning to end*. Harvard University Press.

[CR109] Wuerthner, G., Crist, E., & Butler, T. (2015). *Protecting the wild: Parks and wilderness, the foundation for conservation*. Island Press.

[CR110] Zee, J. C. (2020). Machine sky: Social and terrestrial engineering in a Chinese weather system. *American Anthropologist,**122*(1), 9–20. 10.1111/aman.13360

